# Research on domain ontology construction based on the content features of online rumors

**DOI:** 10.1038/s41598-024-62459-4

**Published:** 2024-05-27

**Authors:** Jianbo Zhao, Huailiang Liu, Weili Zhang, Tong Sun, Qiuyi Chen, Yuehai Wang, Jiale Cheng, Yan Zhuang, Xiaojin Zhang, Shanzhuang Zhang, Bowei Li, Ruiyu Ding

**Affiliations:** 1 School of Economics and Management, Xidian University, 266 Xifeng Road, Xi’an, 710071 China; 2 School of Artificial Intelligence, Xidian University, 266 Xifeng Road, Xi’an, 710071 China; 3 School of Telecommunications Engineering, Xidian University, 266 Xifeng Road, Xi’an, 710071 China

**Keywords:** TFI, Rumor content features, Domain ontology, Top-level ontology reuse, New concept discovery, SWRL rules, Computer science, Information technology, Scientific data, Human behaviour, Computational neuroscience, Data acquisition, Data integration, Data mining, Data processing, Databases, Literature mining, Machine learning

## Abstract

Online rumors are widespread and difficult to identify, which bring serious harm to society and individuals. To effectively detect and govern online rumors, it is necessary to conduct in-depth semantic analysis and understand the content features of rumors. This paper proposes a TFI domain ontology construction method, which aims to achieve semantic parsing and reasoning of the rumor text content. This paper starts from the term layer, the frame layer, and the instance layer, and based on the reuse of the top-level ontology, the extraction of core literature content features, and the discovery of new concepts in the real corpus, obtains the core classes (five parent classes and 88 subclasses) of the rumor domain ontology and defines their concept hierarchy. Object properties and data properties are designed to describe relationships between entities or their features, and the instance layer is created according to the real rumor datasets. OWL language is used to encode the ontology, Protégé is used to visualize it, and SWRL rules and pellet reasoner are used to mine and verify implicit knowledge of the ontology, and judge the category of rumor text. This paper constructs a rumor domain ontology with high consistency and reliability.

## Introduction

Online rumors are false information spread through online media, which have the characteristics of wide content^1^, hard to identify^2,3^. Online rumors can mislead the public, disrupt social order, damage personal and collective reputations, and pose a great challenge to the governance of internet information content. Therefore, in order to effectively detect and govern online rumors, it is necessary to conduct an in-depth semantic analysis and understanding of the rumor text content features.

The research on the content features of online rumors focuses on the lexical, syntactic and semantic features of the rumor text, including lexical, syntactic and semantic features^4^, syntactic structure and functional features^5^, source features^5,6^, rhetorical methods^7^, narrative structure^6–8^, language style^6,9,10^, corroborative means^10,11^ and emotional features^10,12–18^. Most of the existing researches on rumor content features are feature mining under a single domain topic type, and lack of mining the influence relationship between multiple features. Therefore, this paper proposes to build an online rumor domain ontology to realize fine-grained hierarchical modeling of the relationship between rumor content features and credible verification of its effectiveness. Domain ontology is a systematic description of the objective existence in a specific discipline^19^. The construction methods mainly include TOVE method^20^, skeleton method^21^, IDEF-5 method^22,23^, methontology method^24,25^ and seven-step method^26,27^, among which seven-step method is the most mature and widely used method at present^28^, which has strong systematicness and applicability^29^, but it does not provide quantitative indicators and methods about the quality and effect of ontology. The construction technology can be divided into the construction technology based on thesaurus conversion, the construction technology based on existing ontology reuse and the semi-automatic and automatic construction technology based on ontology engineering method^30^. The construction technology based on thesaurus conversion and the construction technology based on existing ontology reuse can save construction time and cost, and improve ontology reusability and interoperability, but there are often differences in structure, semantics and scene. Semi-automatic and automatic construction technology based on ontology engineering method The application of artificial intelligence technology can automatically extract ontology elements and structures from data sources with high efficiency and low cost, but the quality and accuracy are difficult to guarantee. Traditional domain ontology construction methods lack effective quality evaluation support, and construction technology lacks effective integration application. Therefore, this paper proposes an improved TFI network rumor domain ontology construction method based on the seven-step method. Starting from the terminology layer, the framework layer and the instance layer, it integrates the top-level ontology and core document content feature reuse technology, the bottom-up semi-automatic construction technology based on N-gram new word discovery algorithm and RoBERTa-Kmeans clustering algorithm, defines the fine-grained features of network rumor content and carries out hierarchical modeling. Using SWRL rules and pellet inference machine, the tacit knowledge of ontology is mined, and the quality of ontology validity and consistency is evaluated and verified.

The structure of this paper is as follows: Sect “Related work” introduces the characteristics of rumor content and the related work of domain ontology construction.; Sect “Research method” constructs the term layer, the frame layer and the instance layer of the domain ontology; Sect “Domain ontology construction” mines and verifies the implicit knowledge of the ontology based on SWRL rules and Pellet reasoner; Sect “Ontology reasoning and validation” points out the research limitations and future research directions; Sect “Discussion” summarizes the research content and contribution; Sect “Conclusion” summarizes the research content and contribution of this paper.

## Related Work

### Content features of online rumors

The content features of online rumors refer to the adaptive description of vocabulary, syntax and semantics in rumor texts. Fu et al.^5^ have made a linguistic analysis of COVID-19’s online rumors from the perspectives of pragmatics, discourse analysis and syntax, and concluded that the source of information, the specific place and time of the event, the length of the title and statement, and the emotions aroused are the important characteristics to judge the authenticity of the rumors; Zhang et al.^6^ summarized the narrative theme, narrative characteristics, topic characteristics, language style and source characteristics of new media rumors; Li et al.^7^ found that rumors have authoritative blessing and fear appeal in headline rhetoric, and they use news and digital headlines extensively, and the topic construction mostly uses programmed fixed structure; Yu et al.^8^ analyzed and summarized the content distribution, narrative structure, topic scene construction and title characteristics of rumors in detail; Mourao et al.^9^ found that the language style of rumors is significantly different from that of real texts, and rumors tend to use simpler, more emotional and more radical discourse strategies; Zhou et al.^10^ analyzed the rumor text based on six analysis categories, such as content type, focus object and corroboration means, and found that the epidemic rumors were mostly “infectious” topics, with narrative expression being the most common, strong fear, and preference for exaggerated and polarized discourse style. Huang et al.^11^ conducted an empirical study based on WeChat rumors, and found that the “confirmation” means of rumors include data corroboration and specific information, hot events and authoritative release; Butt et al.^12^ analyzed the psycholinguistic features of rumors, and extracted four features from the rumor data set: LIWC, readability, senticnet and emotions. Zhou et al.^13^ analyzed the semantic features of fake news content in theme and emotion, and found that the distribution of fake news and real news is different in theme features, and the overall mood, negative mood and anger of fake news are higher; Tan et al.^14^ divided the content characteristics of rumors into content characteristics with certain emotional tendency and social characteristics that affect credibility; Damstra et al.^15^ identified the elements as a consistent indicator of intentionally deceptive news content, including negative emotions causing anger or fear, lengthy sensational headlines, using informal language or swearing, etc. Lai et al.^16^ put forward that emotional rumors can make the rumor audience have similar positive and negative emotions through emotional contagion; Yuan et al.^17^ found that multimedia evidence form and topic shaping are important means to create rumors, which mostly convey negative emotions of fear and anger, and the provision of information sources is related to the popularity and duration of rumors; Ruan et al.^18^ analyzed the content types, emotional types and discourse focus of Weibo’s rumor samples, and found that the proportion of social life rumors was the highest, and the emotional types were mainly hostile and fearful, with the focus on the general public and the personnel of the party, government and military institutions.

The forms and contents of online rumors tend to be diversified and complicated. The existing research on the content features of rumors is mostly aimed at the mining of content characteristics under specific topics, which cannot cover various types of rumor topics, and lacks fine-grained hierarchical modeling of the relationship between features and credible verification of their effectiveness.

### Domain ontology construction

Domain ontology is a unified definition, standardized organization and visual representation of the concepts of knowledge in a specific domain^31,32^, and it is an important source of information for knowledge-based systems^19,33^. Theoretical methods include TOVE method^20^, skeleton method^21^, IDEF-5 method^22,23^, methontology method^24,25^ and seven-step method^26,27^. TOVE method transforms informal description into formal ontology, which is suitable for fields that need accurate knowledge, but it is complex and time-consuming, requires high-level domain knowledge and is not easy to expand and maintain. Skeleton method forms an ontology skeleton by defining the concepts and relationships of goals, activities, resources, organizations and environment, which can be adjusted according to needs and is suitable for fields that need multi-perspective and multi-level knowledge, but it lacks formal semantics and reasoning ability. Based on this method, Ran et al.^34^ constructed the ontology of idioms and allusions. IDEF5 method uses chart language and detailed description language to construct ontology, formalizes and visualizes objective knowledge, and is suitable for fields that need multi-source data and multi-participation, but it lacks a unified ontology representation language. Based on this method, Li et al.^35^ constructed the business process activity ontology of military equipment maintenance support, and Song et al.^36^ established the air defense and anti-missile operation process ontology. Methontology is a method close to software engineering. It systematically develops ontologies through the processes of specification, knowledge acquisition, conceptualization, integration, implementation, evaluation and document arrangement, which is suitable for fields that need multi-technology and multi-ontology integration, but it is too complicated and tedious, and requires a lot of resources and time^37^. Based on this method, Yang et al.^38^ completed the ontology of emergency plan, Duan et al.^39^ established the ontology of high-resolution images of rural residents, and Chen et al.^40^ constructed the corpus ontology of Jiangui. Seven-step method is the most mature and widely used method at present^28^. It is systematic and applicable to construct ontology by determining its purpose, scope, terms, structure, attributes, limitations and examples^29^, but it does not provide quantitative indicators and methods about the quality and effect of ontology. Based on this method, Zhu et al.^41^ constructed the disease ontology of asthma, Li et al.^42^ constructed the ontology of military events, the ontology of weapons and equipment and the ontology model of battlefield environment, and Zhang et al.^43^ constructed the ontology of stroke nursing field, and verified the construction results by expert consultation.

Domain ontology construction technology includes thesaurus conversion, existing ontology reuse and semi-automatic and automatic construction technology based on ontology engineering method^30^. The construction technology based on thesaurus transformation takes the existing thesaurus as the knowledge source, and transforms the concepts, terms and relationships in the thesaurus into the entities and relationships of domain ontology through certain rules and methods, which saves the time and cost of ontology construction and improves the quality and reusability of ontology. However, it is necessary to solve the structural and semantic differences between thesaurus and ontology and adjust and optimize them according to the characteristics of different fields and application scenarios. Wu et al.^44^ constructed the ontology of the natural gas market according to the thesaurus of the natural gas market and the mapping of subject words to ontology, and Li et al.^45^ constructed the ontology of the medical field according to the Chinese medical thesaurus. The construction technology based on existing ontology reuse uses existing ontologies or knowledge resources to generate new domain ontologies through modification, expansion, merger and mapping, which saves time and cost and improves the consistency and interoperability of ontologies, but it also needs to solve semantic differences and conflicts between ontologies. Chen et al.^46^ reuse the top-level framework of scientific evidence source information ontology (SEPIO) and traditional Chinese medicine language system (TCMLS) to construct the ontology of clinical trials of traditional Chinese medicine, and Xiao et al.^47^ construct the domain ontology of COVID-19 by extracting the existing ontology and the knowledge related to COVID-19 in the diagnosis and treatment guide. Semi-automatic and automatic construction technology based on ontology engineering method semi-automatically or automatically extracts the elements and structures of ontology from data sources by using natural language processing, machine learning and other technologies to realize large-scale, fast and low-cost domain ontology construction^48^, but there are technical difficulties, the quality and accuracy of knowledge extraction can not be well guaranteed, and the quality and consistency of different knowledge sources need to be considered. Suet al.^48^ used regular templates and clustering algorithm to construct the ontology of port machinery, Zheng et al.^49^ realized the automatic construction of mobile phone ontology through LDA and other models, Dong et al.^50^ realized the automatic construction of ontology for human–machine ternary data fusion in manufacturing field, Linli et al.^51^ proposed an ontology learning algorithm based on hypergraph, and Zhai et al.^52^ learned from it through part-of-speech tagging, dependency syntax analysis and pattern matching.

At present, domain ontology construction methods are not easy to expand, lack of effective quality evaluation support, lack of effective integration and application of construction technology, construction divorced from reality can not guide subsequent practice, subjective ontology verification and so on. Aiming at the problems existing in the research of content characteristics and domain ontology construction of online rumors, this paper proposes an improved TFI network rumor domain ontology construction method based on seven-step method, which combines top-down existing ontology reuse technology with bottom-up semi-automatic construction technology, and establishes rumor domain ontology based on top-level ontology reuse, core document content feature extraction and new concept discovery in the real corpus from the terminology layer, framework layer and instance layer. Using Protégé as a visualization tool, the implicit knowledge mining of ontology is carried out by constructing SWRL rules to verify the semantic parsing ability and consistency of domain ontology.

## Research method

This paper proposes a TFI online rumor domain ontology construction method based on the improvement of the seven-step method, which includes the term layer, the frame layer and the instance layer construction.

### Term layer construction

Determine the domain and scope: the purpose of constructing the rumor domain ontology is to support the credible detection and governance of online rumors, and the domain and scope of the ontology are determined by answering questions.

Three-dimensional term set construction: investigate the top-level ontology and related core literature, complete the mapping of reusable top-level ontology and rumor content feature concept extraction semi-automatically from top to bottom; establish authoritative real rumor datasets, and complete the domain new concept discovery automatically from bottom to top; based on this, determine the term set of the domain ontology.

### Frame layer construction

Define core classes and hierarchical relationships: combine the concepts of the three-dimensional rumor term set, based on the data distribution of the rumor dataset, define the parent class, summarize the subclasses, design hierarchical relationships and explain the content of each class.

Define core properties and facets of properties: in order to achieve deep semantic parsing of rumor text contents, define object properties, data properties and property facets for each category in the ontology.

### Instance layer construction

Create instances: analyze the real rumor dataset, extract instance data, and add them to the corresponding concepts in the ontology.

Encode and visualize ontology: use OWL language to encode ontology, and use Protégé to visualize ontology, so that ontology can be understood and operated by computer.

Ontology verification: use SWRL rules and pellet reasoner to mine implicit knowledge of ontology, and verify its semantic parsing ability and consistency.

### Ethical statements

This article does not contain any studies with human participants performed by any of the authors.

## Domain ontology construction

### Term layer construction

#### Determine the professional domain and scope of the ontology description

This paper determines the domain and scope of the online rumor domain ontology by answering the following four questions:

- (1) What is the domain covered by the ontology?

The “Rumor Domain Ontology” constructed in this paper only considers content features, not user features and propagation features; the data covers six rumor types of politics and military, disease prevention and treatment, social life, science and technology, nutrition and health, and others involved in China’s mainstream internet rumor-refuting websites.

- (2) What is the purpose of the ontology?

To perform fine-grained hierarchical modeling of the relationships among the features of multi-domain online rumor contents, realize semantic parsing and credibility reasoning verification of rumor texts, and guide fine-grained rumor detection and governance. It can also be used as a guiding framework and constraint condition for online rumor knowledge graph construction.

- (3) What kind of questions should the information in the ontology provide answers for?

To provide answers for questions such as the fine-grained rumor types of rumor instances, the valid features of rumor types, etc.

- (4) Who will use the ontology in the future?

Users of online rumor detection and governance, users of online rumor knowledge graphs construction.

#### Three-dimensional term set construction

##### Domain concepts reused by top-level ontology

As a mature and authoritative common ontology, top-level ontology can be shared and reused in a large range, providing reference and support for the construction of domain ontology. The domain ontology of online rumors established in this paper focuses on the content characteristics, mainly including the content theme, events and emotions of rumor texts. By reusing the terminology concepts in the existing top-level ontology, the terminology in the terminology set can be unified and standardized. At the same time, the top-level concept and its subclass structure can guide the framework construction of domain ontology and reduce the difficulty and cost of ontology construction. Reusable top-level ontologies include: SUMO, senticnet and ERE after screening.

SUMO ontology: a public upper-level knowledge ontology containing some general concepts and relations for describing knowledge in different domains. The partial reusable SUMO top-level concepts and subclasses selected in this paper are shown in Table 1, which provides support for the sub-concept design of text topics in rumor domain ontology.

**Table 1:** Explanation of some reusable SUMO top-level concepts and examples of subclasses.

Number	Top-level concept	Explanation	Examples of subclass
1	Political process	A policy domain that involves aspects of international security, security assistance, military operations, defense strategy and policy, military space utilization, and defense trade	International relations, security cooperation, military strategy, military technology, military law, etc
2	Health status	A scientific domain that involves the relationship between food, nutrition, body and health	Diet, nutrition, food safety, malnutrition, dietary supplement, etc
3	Prevention	The prevention and control of infectious diseases	Immunization, infection control, infection prevention, etc
4	Science	A specific branch of scientific knowledge natural	Natural science, social science, formal science
5	Credibility	The degree to which a person or thing is considered credible or trustworthy	Synonyms: credibility, believability, etc

Senticnet: a knowledge base for concept-based sentiment analysis, which contains semantic, emotional, and polarity information related to natural language concepts. The partial reusable SenticNet top-level concepts and subclasses selected in this paper are shown in Table 2, which provides support for the sub-concept design of text topics in rumor domain ontology.

**Table 2:** Explanation of some reusable SenticNet top-level concepts and examples of subclasses.

Number	Top-level concept	Explanation	Examples of subclass
1	Polarity value	Indicate the polarity value of the emotion	Negative, positive
2	Primary emotion	A category that represents the basic emotions of a concept,	Sadness, grief, anxiety, annoyance, contentment, melancholy, dislike, etc
3	Seconday emotion	A more complex emotion that is composed or derived from primary emotions	Loathing, acceptance, delight, acceptance, dislike, pleasantness, disgust, etc

Entities, relations, and events (ERE): a knowledge base of events and entity relations. The partial reusable ERE top-level concepts and subclasses selected in this paper are shown in Table 3, which provides support for the sub-concept design of text elements in the rumor domain ontology.

**Table 3:** Explanation of some reusable ERE top-level concepts and examples of subclasses.

Number	Top-level concept	Explanation	Examples of subclass
1	Life	A event that describes the birth of a person or an animal.This item is related to health and nutrition, disease prevention and treatment, and social life	Be-born, marry, divorce, injure, die, etc
2	Business	An event that is related to social life	Start-org, merge-org, declare-bankruptc, end-org, etc
3	Justice	A event that is related to politics and military, major political events, and rumor credibility	Arrest-jail, release-parole, trial-hearing, charge-indict, sue, convict, sentence, fine, execute, extradite, acquit, pardo, appeal, etc
4	Person	A human individual who has an identity. This item is related to the content element of the character	Individual, title, role, etc
5	Organization	An entity that consists of multiple people or other organizations, and has a common goal, function, or characteristic. This item is related to the content element of the institution	Government, commercial, educational, non-governmental, religious, sports

##### Extracting domain concepts based on core literature content features

Domain core literature is an important source for extracting feature concepts. This paper uses ‘rumor detection’ as the search term to retrieve 274 WOS papers and 257 CNKI papers from the WOS and CNKI core literature databases. The content features of rumor texts involved in the literature samples are extracted, the repetition content features are eliminated, the core content features are screened, and the canonical naming of synonymous concepts from different literatures yields the domain concepts as shown in Table 4. Among them, text theme, text element, text style, text feature and text rhetoric are classified as text features; emotional category, emotional appeal and rumor motive are classified as emotional characteristics; source credibility, evidence credibility and testimony method are classified as information credibility characteristics; social context is implicit.

**Table 4:** Part of rumor domain concepts based on literature content features extraction.

Rumor feature	Literature source
Text theme	Zhou et al.(2023)^13^;Zhou et al.(2021)^10^;Song et al.(2020)^55^;Du et al.(2019)^54^;Yu et al.(2018)^8^;Duan et al.(2016)^53^; Zhang et al.(2016)^6^;Huang et al.(2015)^57^;Ruan et al.(2014)^18^;Jiang et al.(2011)^56^
Text element	Damstra et al.(2021)^15^
Text style	Zhou et al.(2021)^10^;Li et al.(2018)^7^
Text feature	Li et al.(2018)^7^;Liu et al.(2018)^58^
Text rhetoric	Damstra et al.(2021)^15^
Emotion category	Tang et al.(2021)^61^;Zhou et al.(2021)^10^;Dong et al.(2020)^59^;Zeng et al.(2019)^60^;Yuan et al.(2015)^17^
Emotional appeal	Pröllochs et al.(2021)^62^;Lai et al.(2016)^16^;Deng et al.(2005)^63^
rumor motive	Ji et al.(2019)^64^;Yuan et al.(2016)^65^;Yuan et al.(2015)^17^;Zhao et al.(2013)^66^
Source credibility	Li et al.(2018)^7^;Yuan et al.(2015)^17^
Evidence credibility	Tang et al.(2021)^61^
Testimony method	Zhou et al.(2021)^10^;Huang et al.(2018)^11^;Yuan et al.(2015)^17^
Social context	Yuan et al.(2023)^68^;Hu et al.(2012)^67^;Wang et al.(2012)^69^;Deng et al.(2005)^63^

##### Extracting domain concepts based on new concept discovery

This paper builds a general rumor dataset based on China’s mainstream rumor-refuting websites as data sources, and proposes a domain new concept discovery algorithm to discover domain new words in the dataset, add them to the word segmentation dictionary to improve the accuracy of word segmentation, and cluster them according to rumor type, resulting in a concept subclass dictionary based on the real rumor dataset, which provided realistic basis and data support for the conceptual design of each subclass in domain ontology.

##### Building a general rumor dataset

The rumor dataset constructed in this paper contains 12,472 texts, with 6236 rumors and 6236 non-rumors; the data sources are China’s mainstream internet rumor-refuting websites: 1032 from the internet rumor exposure platform of China internet joint rumor-refuting platform, 270 from today’s rumor-refuting of China internet joint rumor-refuting platform, 1852 from Tencent news Jiaozhen platform, 1744 from Baidu rumor-refuting platform, 7036 from science rumor-refuting platform, and 538 from Weibo community management center. This paper invited eight researchers to annotate the labels (rumor, non-rumor), categories (politics and military, disease prevention and treatment, social life, science and technology, nutrition and health, others) of the rumor dataset. Because data annotation is artificial and subjective, in order to ensure the effectiveness and consistency of annotation, before inviting researchers to annotate, this paper formulates annotation standards, including the screening method, trigger words and sentence break identification of rumor information and corresponding rumor information, and clearly explains and exemplifies the screening method and trigger words of rumor categories, so as to reduce the understanding differences among researchers; in view of this standard, researchers are trained in labeling to familiarize them with labeling specifications, so as to improve their labeling ability and efficiency. The method of multi-person cross-labeling is adopted when labeling, and each piece of data is independently labeled by at least two researchers. In case of conflicting labeling results, the labeling results are jointly decided by the data annotators to increase the reliability and accuracy of labeling. After labeling, multi-person cross-validation method is used to evaluate the labeling results. Each piece of data is independently verified by at least two researchers who did not participate in labeling, and conflicting labeling results are jointly decided by at least five researchers to ensure the consistency of evaluation results. Examples of the results are shown in Table 5.

**Table 5:** Examples of the rumor dataset annotation.

Number	Text content	Category	Label
1	Shi Zhengli of Wuhan Institute of Virology fled to the US embassy to seek asylum	Politics and military	Rumor
2	Shi Zhengli posted on her WeChat Moments saying: “Dear friends, I and my family are all well! No matter how difficult it is, there will be no ‘defection’ as the rumor says”	Politics and military	Non-rumor
3	Bee venom can inhibit the new coronavirus	Disease prevention and treatment	Rumor
4	Bee venom has a certain degree of neurotoxicity and hematotoxicity. If someone eats bee venom on their own to prevent COVID-19, they may cause poisoning, allergic reactions and other consequences, and even endanger their lives in severe cases	Disease prevention and treatment	Non-rumor
5	A man lost 18 million yuan in gambling and jumped off a building	Social life	Rumor
6	According to the investigation, the man had suicidal thoughts due to illness. The public security authorities have initially ruled out the possibility of criminal cases	Social life	Non-rumor
7	The “space charging” technology produces extremely high levels of radiation, which can be hazardous to your health	Science and technology	Rumor
8	When discussing the impact of radiation on human body, radiation dose must be considered. If the charging power of electronic products is not high, the impact on human body can be ignored	Science and technology	Non-rumor
9	Old cured meat is like old wine, the aroma will be stronger	Nutrition and health	Rumor
10	Bacon has a shelf life, too, and it’s not better the longer you store it	Nutrition and health	Non-rumor

##### N-gram word granularity rumor text new word discovery algorithm

**Figure 1:**
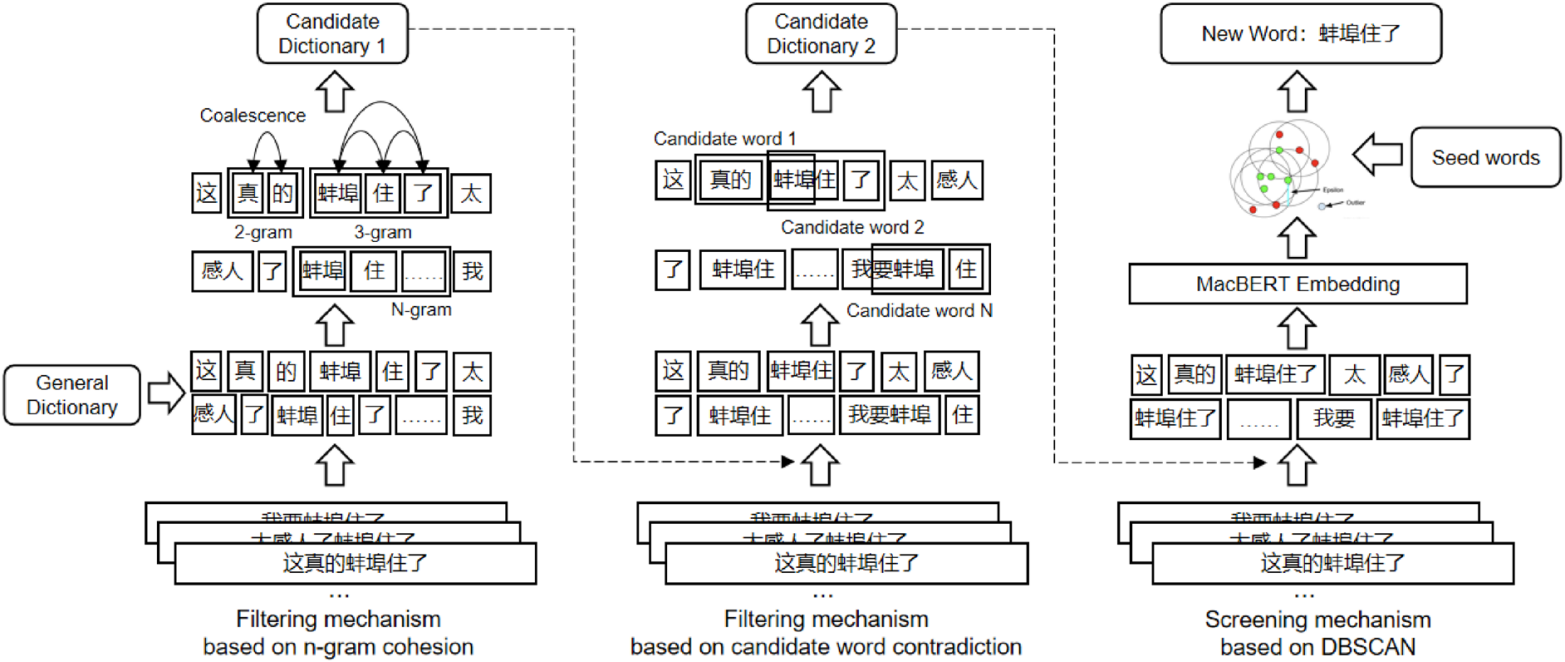
Flowchart of domain new word discovery algorithm.

Existing neologism discovery algorithms are mostly based on the granularity of Chinese characters, and the time complexity of long word discovery is high and the accuracy rate is low. The algorithm’s usefulness is low, and the newly discovered words are mostly already found in general domain dictionaries. To solve these problems, this paper proposes an online rumor new word discovery algorithm based on N-gram word granularity, as shown in Fig. 1.

First, obtain the corpus to be processed ${\varvec{c}}=\{{{\varvec{s}}}_{1},{{\varvec{s}}}_{2},...,{{\varvec{s}}}_{{{\varvec{n}}}_{{\varvec{c}}}}\}$ , and perform the first preprocessing on the corpus to be processed, which includes: sentence segmentation, Chinese word segmentation and punctuation removal for the corpus to be processed. Obtain the first corpus ${{\varvec{c}}}^{{\varvec{p}}}=\{{{\varvec{s}}}_{1}^{{\varvec{p}}},{{\varvec{s}}}_{2}^{{\varvec{p}}},...,{{\varvec{s}}}_{{{\varvec{n}}}_{{\varvec{c}}}}^{{\varvec{p}}}\}$ ; where ${s}_{i}$ represents the $i$-th sentence in the corpus to be processed, ${n}_{c}$ represents the number of sentences in the corpus to be processed, and ${s}_{i}^{p}$ is the i-th sentence in the first corpus; perform N-gram operation on each sentence in the first corpus separately, and obtain multiple candidate words $n=2\sim 5$; count the word frequency of each candidate word in the first corpus, and remove the candidate words with word frequency less than the first threshold, and obtain the first class of candidate word set;calculate the cohesion of each candidate word in the first class of candidate word set according to the following formula:

1 $${\varvec{min}}\left\{ {\frac{{{\varvec{P}}\left( {{\varvec{g}}_{1} {\varvec{g}}_{2} {\varvec{g}}_{3} {\varvec{g}}_{4} } \right)}}{{{\varvec{P}}\left( {{\varvec{g}}_{1} } \right){\varvec{P}}\left( {{\varvec{g}}_{2} {\varvec{g}}_{3} {\varvec{g}}_{4} } \right)}},\frac{{{\varvec{P}}\left( {{\varvec{g}}_{1} {\varvec{g}}_{2} {\varvec{g}}_{3} {\varvec{g}}_{4} } \right)}}{{{\varvec{P}}\left( {{\varvec{g}}_{1} {\varvec{g}}_{2} } \right){\varvec{P}}\left( {{\varvec{g}}_{3} {\varvec{g}}_{4} } \right)}},\frac{{{\varvec{P}}\left( {{\varvec{g}}_{1} {\varvec{g}}_{2} {\varvec{g}}_{3} {\varvec{g}}_{4} } \right)}}{{{\varvec{P}}\left( {{\varvec{g}}_{1} {\varvec{g}}_{2} {\varvec{g}}_{3} } \right){\varvec{P}}\left( {{\varvec{g}}_{4} } \right)}}} \right\}$$

In the formula, $P(\cdot )$ represents word frequency.Then filter according to the second threshold corresponding to N-gram operation, and obtain the second class of candidate word set; after loading the new words in the second class of candidate word set into LTP dictionary, perform the second preprocessing on the corpus to be processed ${\varvec{c}}=\{{{\varvec{s}}}_{1},{{\varvec{s}}}_{2},...,{{\varvec{s}}}_{{{\varvec{n}}}_{{\varvec{c}}}}\}$; and obtain the second corpus ${{\varvec{c}}}^{{\varvec{p}}\boldsymbol{^{\prime}}}=\{{{\varvec{s}}}_{1}^{{\varvec{p}}\boldsymbol{^{\prime}}},{{\varvec{s}}}_{2}^{{\varvec{p}}\boldsymbol{^{\prime}}},...,{{\varvec{s}}}_{{{\varvec{n}}}_{{\varvec{c}}}}^{{\varvec{p}}\boldsymbol{^{\prime}}}\}$; where the second preprocessing includes: sentence segmentation, Chinese word segmentation and stop word removal for the corpus to be processed; after obtaining the vector representation of each word in the second corpus, determine the vector representation of each new word in the second class of candidate word set; according to the vector representation of each new word, use K-means algorithm for clustering; according to the clustering results and preset classification rules, classify each new word to the corresponding domain. The examples of new words discovered are shown in Table 6:

**Table 6:** Examples of domain neologism discovery.

Number	Category	New words discovered
1	Politics and military	Lockdown, Sino-Soviet, CPPCC members, Chinese government, wish for world peace, etc
2	Disease prevention and treatment	Drinking water, calcium supplementation, medication, sterilization, nucleic acid testing, COVID-19 patients, etc
3	Social life	Wearing masks, being isolated, online, going to college, latest news, etc
4	Science and technology	Spontaneous fever, APP monitoring, enamel products, asteroid hitting the earth, etc
5	Nutrition and health	O blood type, sugar-free drinks, nutritional value, dietary fiber, osteoporosis, etc

##### RoBERTa-Kmeans rumor text concepts extraction algorithm

After adding the new words obtained by the new word discovery to the LTP dictionary, the accuracy of LTP word segmentation is improved. The five types of rumor texts established in this paper are segmented by using the new LTP dictionary, and the word vectors are obtained by inputting them into the RoBERTa word embedding layer after removing the stop words. The word vectors are clustered by k-means according to rumor type to obtain the concept subclass dictionary. The main process is as follows:

- (1) Word embedding layer

The RoBERTa model uses Transformer-Encode for computation, and each module contains multi-head attention mechanism, residual connection and layer normalization, feed-forward neural network. The word vectors are obtained by representing the rumor texts after accurate word segmentation through one-hot encoding, and the position encoding represents the relative or absolute position of the word in the sequence. The word embedding vectors generated by superimposing the two are used as input X. The multi-head attention mechanism uses multiple independent Attention modules to perform parallel operations on the input information, as shown in formula (2):

2 $${\varvec{Attention}}\left( {{\varvec{Q}},{\varvec{K}},{\varvec{V}}} \right) = {\varvec{Softmax}}\left( {\frac{{{\varvec{QK}}^{{\varvec{T}}} }}{{\sqrt {{\varvec{d}}_{{\varvec{k}}} } }}} \right){\varvec{V}}$$

where $\left\{{\varvec{Q}},{\varvec{K}},{\varvec{V}}\right\}$ is the input matrix, ${{\varvec{d}}}_{{\varvec{k}}}$ is the dimension of the input matrix. After calculation, the hidden vectors obtained after computation are residual concatenated with layer normalization, and then calculated by two fully connected layers of feed-forward neural network for input, as shown in formula (3):

3 $${\varvec{W}}_{{\varvec{e}}} = {\varvec{max}}\left( {0,{\varvec{XW}}_{0} + {\varvec{b}}_{0} } \right){\varvec{W}}_{0} \user2{^{\prime}} + \user2{b^{\prime}}$$

where $\left\{{{\varvec{W}}}_{{\varvec{e}}},{{\varvec{W}}}_{0}\boldsymbol{^{\prime}}\right\}$ are the weight matrices of two connected layers, $\left\{{{\varvec{b}}}_{{\varvec{e}}},{{\varvec{b}}}_{0}\boldsymbol{^{\prime}}\right\}$ are the bias terms of two connected layers.

After calculation, a bidirectional association between word embedding vectors is established, which enables the model to learn the semantic features contained in each word embedding vector in different contexts. Through fine-tuning, the learned knowledge is transferred to the downstream clustering task.

- (2) K-means clustering

Randomly select k initial points to obtain k classes, and iterate until the loss function of the clustering result is minimized. The loss function can be defined as the sum of squared errors of each sample point from its cluster center point, as shown in formula (4).

4 $${\varvec{L}}\left( {{\varvec{\alpha}},{\varvec{\mu}}} \right) = \mathop \sum \limits_{{{\varvec{i}} = 1}}^{{\varvec{N}}} \parallel{\varvec{x}}_{{\varvec{i}}} - {\varvec{u}}_{{{\varvec{a}}_{{\varvec{i}}} }}\parallel^{2}$$

where ${x}_{i}$ represents the $i$ sample, ${a}_{i}$ is the cluster that ${x}_{i}$ belongs to, ${u}_{{a}_{i}}$ represents the corresponding center point, $N$ is the total number of samples.

After RoBERTa-kmeans calculation, the concept subclasses obtained are manually screened, merged repetition items, deleted invalid items, and finally obtained 79 rumor concept subclasses, including 14 politics and military subclasses, 23 disease prevention and treatment subclasses, 15 social life subclasses, 13 science and technology subclasses, and 14 nutrition and health subclasses. Some statistics are shown in Table 7.

**Table 7:** Rumor Theme Partial Concept Subcategory Statistics.

Number	Category	Conceptual subcategories extracted
1	Politics and military	Major event, military and political figure, social security, international, etc
2	Disease prevention and treatment	Body parts, epidemic prevention and control, chemicals, hazardous materials, etc
3	Social life	Daily life, education, transportation, family, etc
4	Science and technology	Astronomy, agriculture, ocean, computers, etc
5	Nutrition and health	Diet, vegetables and fruits, exercise and health, bacteria and viruses, etc

Each concept subclass is obtained by clustering several topic words. For example, the topic words that constitute the subclasses of body part, epidemic prevention and control, chemical drugs, etc. under the disease prevention and treatment topic are shown in Table 8.

- (3) Determining the terminology set

**Table 8:** Statistics of some conceptual subcategories and their subject terms under the theme of disease prevention and treatment.

Number	Concept subclass	Topic words
1	Body part	Arm, skin, kidney, throat, mucosa…
2	Epidemic prevention and control	Disinfection, prevention, epidemic prevention, cabin, gathering…
3	Chemical drug	Hydroxychloroquine, levofloxacin, quercetin…

This paper constructs a three-dimensional rumor domain ontology terminology set based on the above three methods, and unifies the naming of the terms. Some of the terms are shown in Table 9.

**Table 9:** Examples of three-dimensional rumor domain ontology terminology set.

Source dimension	Ontology terminology
Domain concepts for top-level ontology reuse	Science, technology, emotion, person, organization, location, prevention, political process, health status, credibility, polarity value, primary emotion…
Extracting domain concepts based on core literature content features	Text theme, text element, text style, text feature, text rhetoric, emotion category, emotional appeal, rumor motive, source credibility, evidence credibility, testimony method, social context…
Extract domain concepts based on new concept discovery	Major event, military and political figure, social security, epidemic prevention and control, chemical drug, hazardous substance, daily, education, transportation, astronomy, agriculture and ocean, computer, diet, vegetables and fruit, sports and health, bacteria and viruses…

### Framework layer construction

#### Define core classes and hierarchy

##### Define parent classes

This paper aims at fine-grained hierarchical modeling of the relationship between the content characteristics of multi-domain network rumors. Therefore, the top-level parent class needs to include the rumor category and the main content characteristics of a sub-category rumor design. The main content characteristics are the clustering results of domain concepts extracted based on the content characteristics of core documents, that is, rumor text feature, rumor emotional characteristic, rumor credibility and social context. The specific contents of the five top parent classes are as follows:

Rumor type: the specific classification of rumors under different subject categories; Rumor text feature, the common features of rumor texts in terms of theme, style, rhetoric, etc. Rumor emotional characteristic: the emotional elements of rumor texts, the Rumor motive of the publisher, and the emotional changes they hope to trigger in the receiver. Rumor credibility: the authority of the information source, the credibility of the evidence material provided by the publisher, and the effectiveness of the testimony method. Social context: the relevant issues and events in the society when the rumor is published.

##### Induce subclasses and design hierarchical relationships

**Figure 2:**
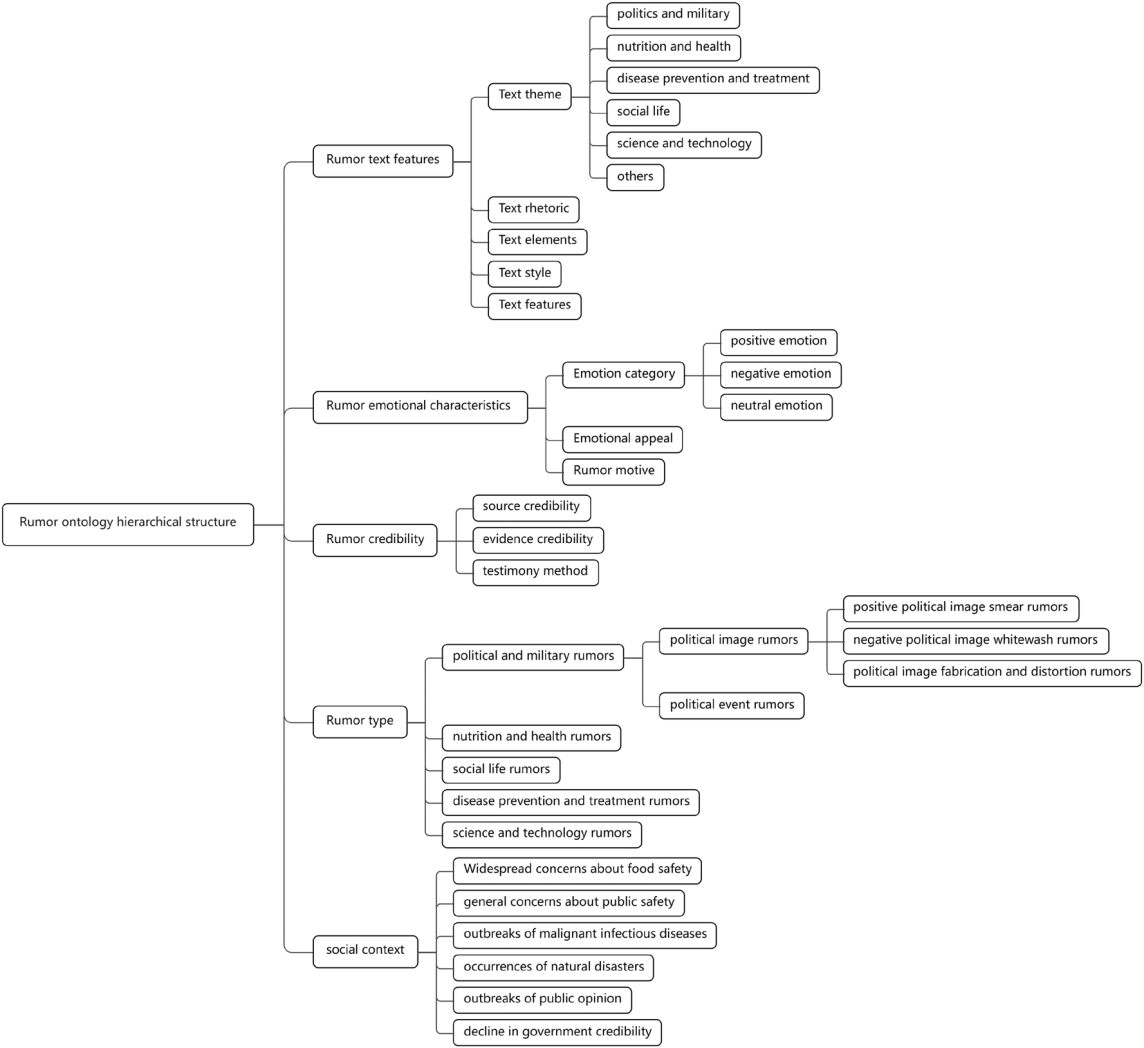
Diagram of the core classes and hierarchy of the rumor domain ontology.

In this paper, under the top-level parent class, according to the top-level concepts of top-level ontologies such as SUMO, senticnet and ERE and their subclass structures, and the rumor text features of each category extracted from the real rumor text dataset, we summarize its 88 subclasses and design the hierarchical relationships, as shown in Fig. 2, which include:

- (1) Rumor text feature

①Text theme^6,8,13,18,53^: the theme or topic that the rumor text content involves. Based on the self-built rumor dataset, it is divided into politics and military^54^, involving information such as political figures, political policies, political relations, political activities, military actions, military events, strategic objectives, politics and military reviews, etc.; nutrition and health^55^, involving information such as the relationship between human health and nutrition, the nutritional components and value of food, the plan and advice for healthy eating, health problems and habits, etc.; disease prevention and treatment^10^, involving information such as the definition of disease, vaccine, treatment, prevention, data, etc.; social life^56^, involving information such as social issues, social environment, social values, cultural activities, social media, education system, etc.; science and technology^57^, involving information such as scientific research, scientific discovery, technological innovation, technological application, technological enterprise, etc.; other categories.

②Text element^15^: the structured information of the rumor text contents. It is divided into character, political character, public character, etc.; geographical position, city, region, area, etc.; event, historical event, current event, crisis event, policy event, etc.; action, protection, prevention and control, exercise, fighting, crime, eating, breeding, health preservation, rest, exercise, education, sports, social, cultural, ideological, business, economic, transportation, etc.; material, food, products (food, medicine, health products, cosmetics, etc.) and the materials they contain and their relationship with human health. effect, nutrition, health, harm, natural disaster, man-made disaster, guarantee, prevention, treatment, etc.; institution, government, enterprise, school, hospital, army, police, social group, etc.; nature, weather, astronomy, environment, agriculture, disease, etc.

③Text style^7,10^: the discourse style of the rumor text contents, preferring exaggerated and emotional expression. It is divided into gossip style, creating conflict or entertainment effect; curious style, satisfying people’s curiosity and stimulation; critical style, using receivers’ stereotypes or preconceptions; lyrical style, creating resonance and influencing emotion; didactic style influencing receivers’ thought and behavior from an authoritative perspective; plain style concise objective arousing resonance etc.

④Text feature^7,58^: special language means in the rumor text contents that can increase the transmission and influence of the rumor. It is divided into extensive punctuation reminding or attracting receivers’ attention; many mood words enhancing emotional color and persuasiveness; many emoji conveying attitude; induce forwarding using @ symbol etc. to induce receivers to forward etc.

⑤Text rhetoric^15^: common rhetorical devices in rumor contents. It is divided into metaphor hyperbole repetition personification etc.

- (2) Rumor emotional characteristic

①Emotion category^17,59,60^: the emotional tendency and intensity expressed in the rumor texts. It is divided into positive emotion happy praise etc.; negative emotion fear^10^ anger sadness anxiety^61^ dissatisfaction depression etc.; neutral emotion no preference plain objective etc.

②Emotional appeal^16,62,63^: the online rumor disseminator hopes that the rumor they disseminate can trigger some emotional changes in the receiver. It is divided into “joy” happy pleasant satisfied emotions that prompt receivers to spread or believe some rumors that are conducive to social harmony; “love” love appreciation admiration emotions that prompt receivers to spread or believe some rumors that are conducive to some people or group interests; “anger” angry annoyed dissatisfied emotions that prompt receivers to spread or believe some rumors that are anti-social or intensify conflicts; “fear” fearful afraid nervous emotions that prompt receivers to spread or believe some rumors that have bad effects deliberately exaggerated; “repugnance” disgusted nauseous emotions that prompt receivers to spread or believe some rumors that are detrimental to social harmony; “surprise” surprised shocked amazed emotions that prompt receivers to spread or believe some rumors that deliberately attract traffic exaggerated fabricated etc.

③Rumor motive^17,64–66^: the purpose and need of the rumor publisher to publish rumors and the receiver to forward rumors. Such as profit-driven seeking fame and fortune deceiving receivers; emotional catharsis relieving dissatisfaction emotions by venting; creating panic creating social unrest and riots disrupting social order; entertainment fooling receivers seeking stimulation; information verification digging out the truth of events etc.

- (3) Rumor credibility

①source credibility^7,17^: the degree of trustworthiness that the information source has. Such as official institutions and authoritative experts and scholars in the field with high credibility; well-known encyclopedias and large-scale civil organizations with medium credibility; small-scale civil organizations and personal hearsay personal experience with low credibility etc.

②evidence credibility^61^: the credibility of the information proof material provided by the publisher. Data support such as scientific basis based on scientific theory or method; related feature with definite research or investigation result in data support; temporal background with clear time place character event and other elements which related to the information content; the common sense of life in line with the facts and scientific common sense that are widely recognized.

③testimony method^10,11,17^: the method to support or refute a certain point of view. Such as multimedia material expressing or fabricating content details through pictures videos audio; authority endorsement policy documents research papers etc. of authorized institutions or persons; social identity identity of social relation groups.

- (4) Social context

①social issue^67^: some bad phenomena or difficulties in society such as poverty pollution corruption crime government credibility decline^68^ etc.

②public attention^63^: events or topics that arouse widespread attention or discussion in the society such as sports events technological innovation food safety religious beliefs Myanmar fraud nuclear wastewater discharge etc.

③emergency(public sentiment)^69^: some major or urgent events that suddenly occur in society such as earthquake flood public safety malignant infectious disease outbreaks etc.

- (5) Rumor type

①Political and military rumor:

Political image rumor: rumors related to images closely connected to politics and military, such as countries, political figures, institutions, symbols, etc. These include positive political image smear rumor, negative political image whitewash rumor, political image fabrication and distortion rumor, etc.

Political event rumor: rumors about military and political events, such as international relations, security cooperation, military strategy, judicial trial, etc. These include positive political event smear rumor, negative political event whitewash rumor, political event fabrication and distortion rumor, etc.

②Nutrition and health rumor:

Food product rumor: rumors related to food, products (food, medicine, health products, cosmetics, etc.), the materials they contain and their association with human health. These include positive effect of food product rumor, negative effect of food product rumor, food product knowledge rumor, etc.

Living habit rumor: rumors related to habitual actions in life and their association with human health. These include positive effect of living habit rumor, negative effect of living habit rumor, living habit knowledge rumor, etc.

③Disease prevention and treatment rumor:

Disease management rumor: rumors related to disease management and control methods that maintain and promote individual and group health. These include positive prevention and treatment rumor, negative aggravating disease rumor, disease management knowledge rumor, etc.

Disease confirmed transmission rumor: rumors about the confirmation, transmission, and immunity of epidemic diseases at the social level in terms of causes, processes, results, etc. These include local confirmed cases rumor, celebrity confirmed cases rumor, transmission mechanism rumor, etc.

Disease notification and advice rumor: rumors that fabricate or distort the statements of authorized institutions or experts in the field, and provide false policies or suggestions related to diseases. These include institutional notification rumor, expert advice rumor, etc.

④Social life rumor:

Public figure public opinion rumor: rumors related to public figures’ opinions, actions, private lives, etc. These include positive public figure smear rumor, negative public figure whitewash rumor, public figure life exposure rumor, etc.

Social life event rumor: rumors related to events, actions, and impacts on people's social life. These include positive event sharing rumor, negative event exposure rumor, neutral event knowledge rumor, etc.

Disaster occurrence rumor: rumors related to natural disasters or man-made disasters and their subsequent developments. These include natural disaster occurrence rumor, man-made disaster occurrence rumor, etc.

⑤Science and technology rumor:

Scientific knowledge rumor: rumors related to natural science or social science theories and knowledge. These include scientific theory rumor, scientific concept rumor, etc.

Science and technology application rumor: rumors related to the research and development and practical application of science and technology and related products. These include scientific and technological product rumor, scientific and technological information rumor, etc.

⑥Other rumor: rumors that do not contain elements from the above categories.

#### Definition of core properties and facets of properties

Properties in the ontology are used to describe the relationships between entities or the characteristics of entities. Object properties are relationships that connect two entities, describing the interactions between entities; data properties represent the characteristics of entities, usually in the form of some data type. Based on the self-built rumor dataset, this paper designs object properties, data properties and facets of properties for the parent classes and subclasses of the rumor domain ontology.

##### Object properties

A partial set of object properties is shown in Table 10.

**Table 10:** Partial set of the rumor domain ontology object properties.

Object Property Name	Domain	Range	Description	Object properties faceted
HasTheme	Rumor type	Text theme	Describe the content theme of the rumor text	Symmetry
HasStyle	Rumor type	Text style	Describe the writing style of the rumor text	Symmetry
HasElement	Rumor type	Text element	Describe the elements of the rumor text	Symmetry
HasFeature	Rumor type	Text feature	Describe the textual features of the rumor text	Symmetry
HasRhetoric	Rumor type	Text rhetoric	Describe the rhetorical methods used in the rumor text	Symmetry
HasEmotion	Rumor type	Emotion category	Describe the emotional categories of rumor content	Symmetry
HasMood	Rumor type	Emotional appeal	Describe the emotions that the publisher wants the receiver to feel	Symmetry
HasMotive	Rumor type	Rumor motive	Describe the publisher's motivation for publishing the rumor	Symmetry
HasSource	Rumor type	Source credibility	Describe the authority of the source of the rumor	Symmetry
HasEvidence	Rumor type	Evidence credibility	Describe the credibility of the evidence provided by the publisher	Symmetry
HasSupport	Rumor type	Testimony method	Describe the method of testimony used by the publisher	Symmetry
HasBackground	Rumor type	Social context	Describe the social context in which the rumor was published	symmetry

##### Data attributes

The partial data attribute set is shown in Table 11.

**Table 11:** Partial set of the rumor domain ontology data attributes.

Ontology Terms	Attribute name	Facet			
		Value range	Cardinal number	Default value	Property constraints
Character	Name	String	1	NULL	Functional
	Aliases	String	1–10	NULL	–
	Gender	Male, female, other	1	Other	Functional
	Career	String	1–5	NULL	–
	Nationality	String	1–5	NULL	–
	Polarity value	Positive, negative, medium	1	Medium	Functional
Institution	Name	String	1	NULL	Functional
	Aliases	String	1–10	NULL	–
	Location	String	1	NULL	Functional
	Polarity value	Positive, negative, medium	1	Medium	Functional
Event	Name	String	1	NULL	Functional
	Aliases	String	1–10	NULL	–
	Occurrence time	String	1	NULL	Functional
	Occurrence site	String	1	NULL	Functional
	Polarity value	Positive, negative, medium	1	Medium	Functional

### Instance layer construction

#### Creating instances

Based on the defined core classes and properties, this paper creates instances according to the real rumor dataset. An example is shown in Table 12.

**Table 12:** Examples of the rumor domain ontology.

Rumor text	Object property	Range	Parent class	Class introduction
Lin was domestically abused by Ryohei Kurosawa, with the tearful trail of betrayal and the shadow of gambling hanging over her head. Even if she has tried to divorce, can not be resolved, Taiwan media reports ……	HasTheme	Social life	Rumor type-social life rumor-public figure public opinion rumor-public figure life exposure rumor	This kind of rumor involves the exposure of information about public figures. The rumor-mongers use the high popularity of public figures to attract traffic, and spread unconfirmed private information for entertainment or profit purposes. They satisfy the curiosity of the public while creating social public opinion
	HasStyle	Gossip style		
	HasElement	Artiste-Lin Chi-Ling, Artiste- Kurosawa Ryohei, negative action-domestic violence, civil institution-taiwanese media		
	HasRhetoric	Hyperbole, personification		
	HasEmotion	Negative emotion		
	HasMood	Repugnance, surprise		
	HasMotive	Entertainment, profit-driven		
	HasSource	Small-scale civil organization and personal hearsay		
	HasEvidence	Temporal background, sense of presence		
	HasSupport	Multimedia material, social identity		
Playing with your phone for a long time, especially at night, can cause eye macular disease, leading to macular degeneration, which is like having "eye cancer", completely incurable, and even cause blindness in severe cases	HasTheme	Nutrition and health	Rumor type-nutrition and health rumor-living habit rumor—negative effect of living habit rumor	This kind of rumor involves the negative effects of daily living habits. The publisher often uses the form of popular science or sharing, fabricating so-called scientific evidence, claiming that some living habits will cause some diseases and symptoms, using the inherent impression in the common sense of the public to exaggerate the actual impact, achieving the purpose of deceiving traffic or creating panic
	HasStyle	Plain style		
	HasElement	Action-playing on a cell phone, disease name-macular degeneration of the eye, disease name-cancer, disease symptom-blindness		
	HasRhetoric	Hyperbole, metaphor		
	HasEmotion	Negative emotion		
	HasMood	Fear, surprise		
	HasMotive	Create panic, profit-driven		
	HasSource	Small-scale civil organization and personal hearsay		
	HasEvidence	Sense of presence, common sense of life		
	HasSupport	Social identity		
Shanghai's political and legal system has been exposed to another shocking sex scandal! Before the world-shattering controversy of several judges of the Shanghai High Court visiting prostitutes collectively subsided, some media people broke the news that Ma Huaihai, the public security director of Jinshan District in Shanghai, had been found guilty of more than 2 billion dollars of corruption and murdering a member of the city's People's Congress!	HasTheme	Politics and military	Rumor type- political and military rumor- political image rumor- positive political image smear rumor	This kind of rumor involves the malicious smear of positive characters or national institutions, by using extremely exaggerated language to accuse or fabricate scandals of government officials, reducing the trust of the people in the government
	HasElement	Administrative location-Shanghai, party organs-Supreme People’s Court, government officials-public security chiefs, exemplary delegates-deputies to the National People’s Congress, negative action-prostitution, negative action-corruption, negative action-murder		
	HasStyle	Critical style, gossip style		
	HasRhetoric	Hyperbole		
	HasFeature	Extensive punctuation, many mood words		
	HasEmotion	Negative emotion		
	HasMood	Anger, surprise, fear, repugnance		
	HasMotive	Emotional catharsis, profit-driven		
	HasSource	Small-scale civil organization and personal hearsay		
	HasEvidence	Multimedia material, sense of presence		
	HasSupport	Social identity		
	HasBackground	Corruption		
For cars with a high risk of collision, the high-pressure hydrogen cylinders on hydrogen fuel cell vehicles seem like a potential "bomb", and hydrogen fuel cell vehicles are unsafe	HasTheme	Science and technology	Rumor type- science and technology rumor- science and technology application rumor- scientific and technological product rumor	This kind of rumor involves various science and technology that exist in life. The publisher evaluates the positive or negative effects of existing science and technology, and uses the knowledge gap of the public to output wrong opinions or deepen stereotypes, thereby gaining traffic attention or influencing the market
	HasElement	Things-car, things-bomb, technology products-hydrogen batteries		
	HasStyle	Critical style, didactic style		
	HasRhetoric	Metaphor		
	HasEmotion	Negative emotion		
	HasMood	Surprise, repugnance, fear		
	HasMotive	Profit-driven, createing panic		
	HasSource	Small-scale civil organization and personal hearsay		
	HasSupport	Social identity		
Every day before going out with a cotton swab dipped in some small milled sesame oil, drops into the two nostrils, gently pinch a few times can be, so that you can block all the flu and plague infections	HasTheme	Disease prevention and treatment	Rumor type-disease prevention and treatment rumor-disease management rumor-positive prevention and treatment rumor	This kind of rumor involves the prevention and treatment methods of diseases and symptoms. The publisher often uses the form of popular science or sharing, fabricating so-called scientific evidence, claiming that some products, materials, actions can prevent, cure or alleviate some diseases and symptoms, achieving the purpose of selling products or deceiving traffic
	HasStyle	Plain style, didactic style		
	HasElement	Things—cotton swab, food—scented oil, human organ—nose, positive effect—blocking, disease name—flu, disease name—plague		
	HasRhetoric	Hyperbole		
	HasEmotion	Positive emotion		
	HasMood	Joy, love, surprise		
	HasMotive	Profit-driven		
	HasSource	Small-scale civil organization and personal hearsay		
	HasEvidence	Common sense of the life		
	HasSupport	Social identity		
	HasBackground	Outbreaks of malignant infectious disease		

**Figure 3:**
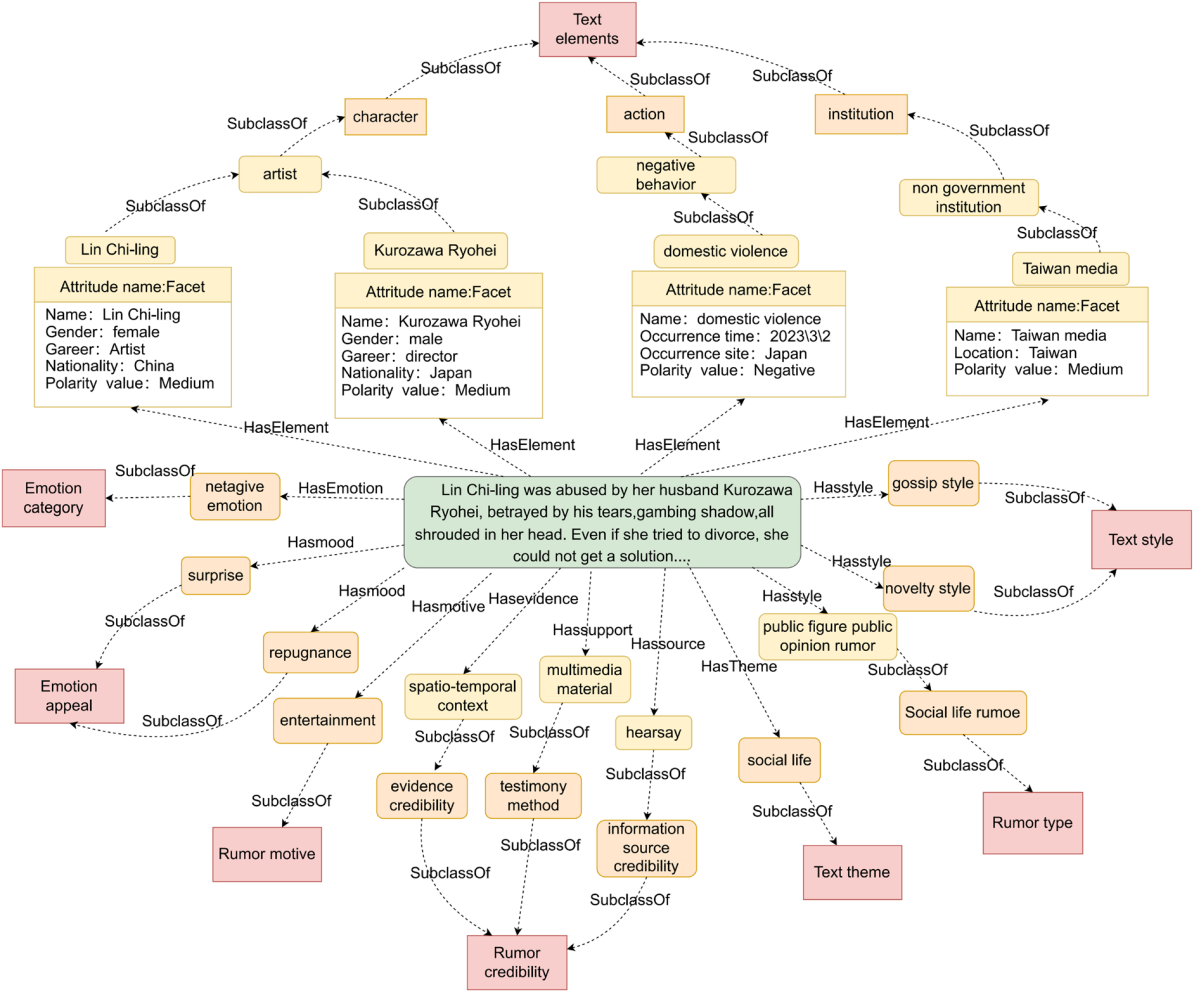
Schematic example of the rumor domain ontology.

This paper selects the online rumor that “Lin Chi-ling was abused by her husband Kuroki Meisa, the tears of betrayal, the shadow of gambling, all shrouded her head. Even if she tried to divorce, she could not get a solution…..” as an example, and draws a structure diagram of the rumor domain ontology instance, as shown in Fig. 3. This instance shows the seven major text features of the rumor text: text theme, text element, text style, emotion category, emotional appeal, rumor motivation, and rumor credibility, as well as the related subclass instances, laying a foundation for building a multi-source rumor domain knowledge graph.

#### Encoding ontology and visualization

##### Encoding ontology

**Figure 4:**
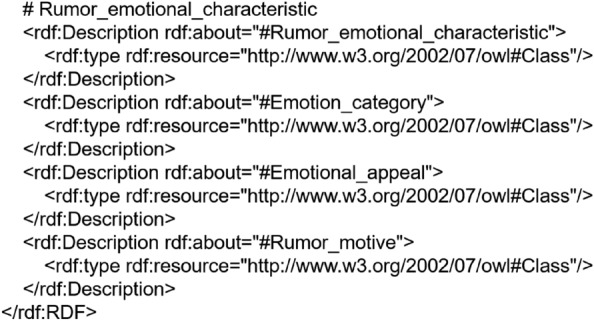
Partial OWL codes of the rumor domain ontology.

This paper uses OWL language to encode the rumor domain ontology, to accurately describe the entities, concepts and their relationships, and to facilitate knowledge reasoning and semantic understanding. Classes in the rumor domain ontology are represented by the class “Class” in OWL and the hierarchical relationship is represented by subclassof. For example, in the creation of the rumor emotional characteristic class and its subclasses, the OWL code is shown in Fig. 4:

The ontology is formalized and stored as a code file using the above OWL language, providing support for reasoning.

##### Ontology visualization

**Figure 5:**
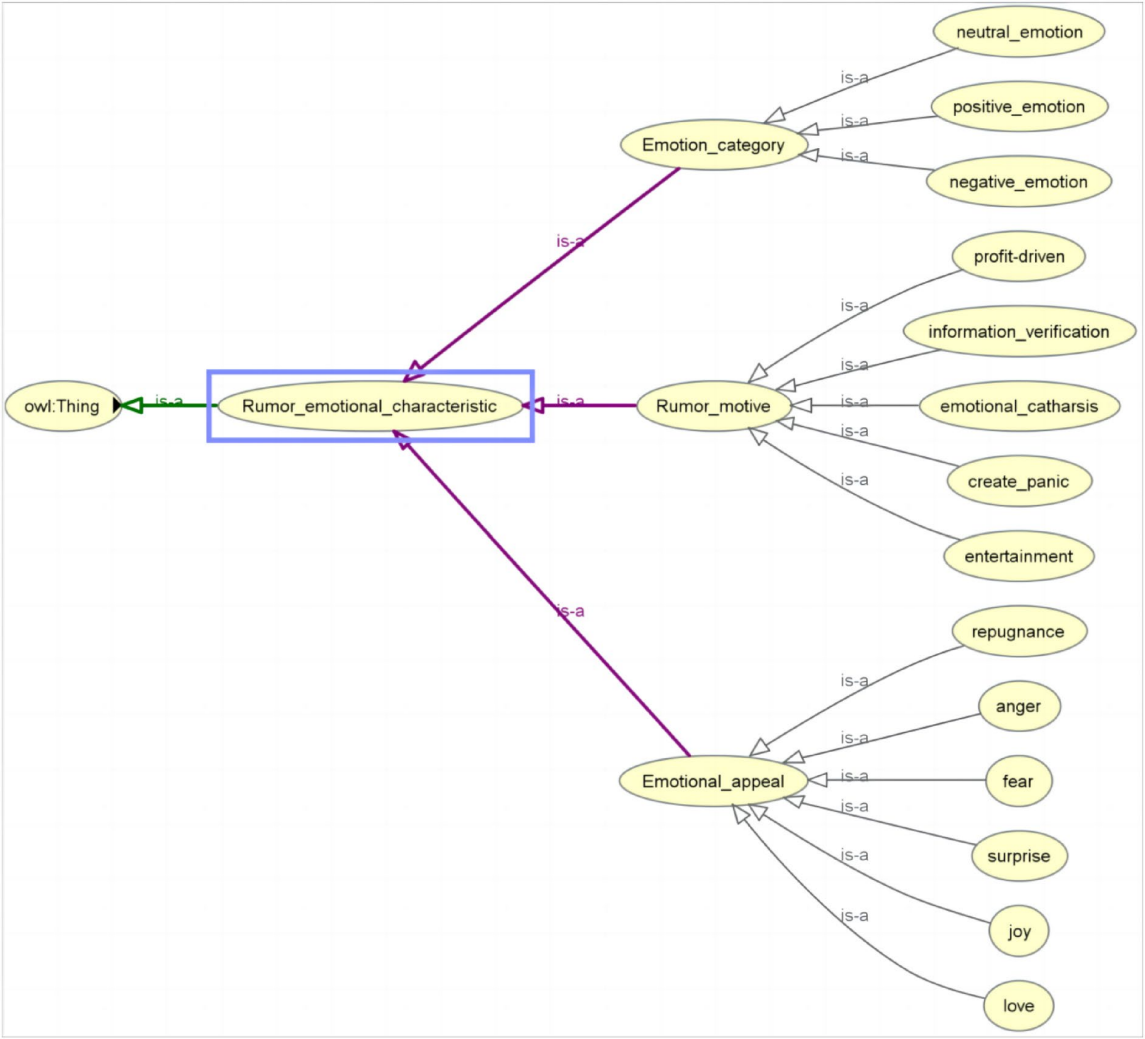
Ontology parent class “RumorEmotionalFeatures” and its subclasses.

This paper uses protégé5.5 to visualize the rumor domain ontology, showing the hierarchical structure and relationship of the ontology parent class and its subclasses. Due to space limitations, this paper only shows the ontology parent class “RumorEmotionalFeatures” and its subclasses, as shown in Fig. 5.

## Ontology reasoning and validation

### SWRL reasoning rule construction

SWRL reasoning rule is an ontology-based rule language that can be used to define Horn-like rules to enhance the reasoning and expressive ability of the ontology. This paper uses SWRL reasoning rules to deal with the conflict relationships between classes and between classes and instances in the rumor domain ontology, and uses pellet reasoner to deeply mine the implicit semantic relationships between classes and instances, to verify the semantic parsing ability and consistency of the rumor domain ontology.

**Figure 6:**
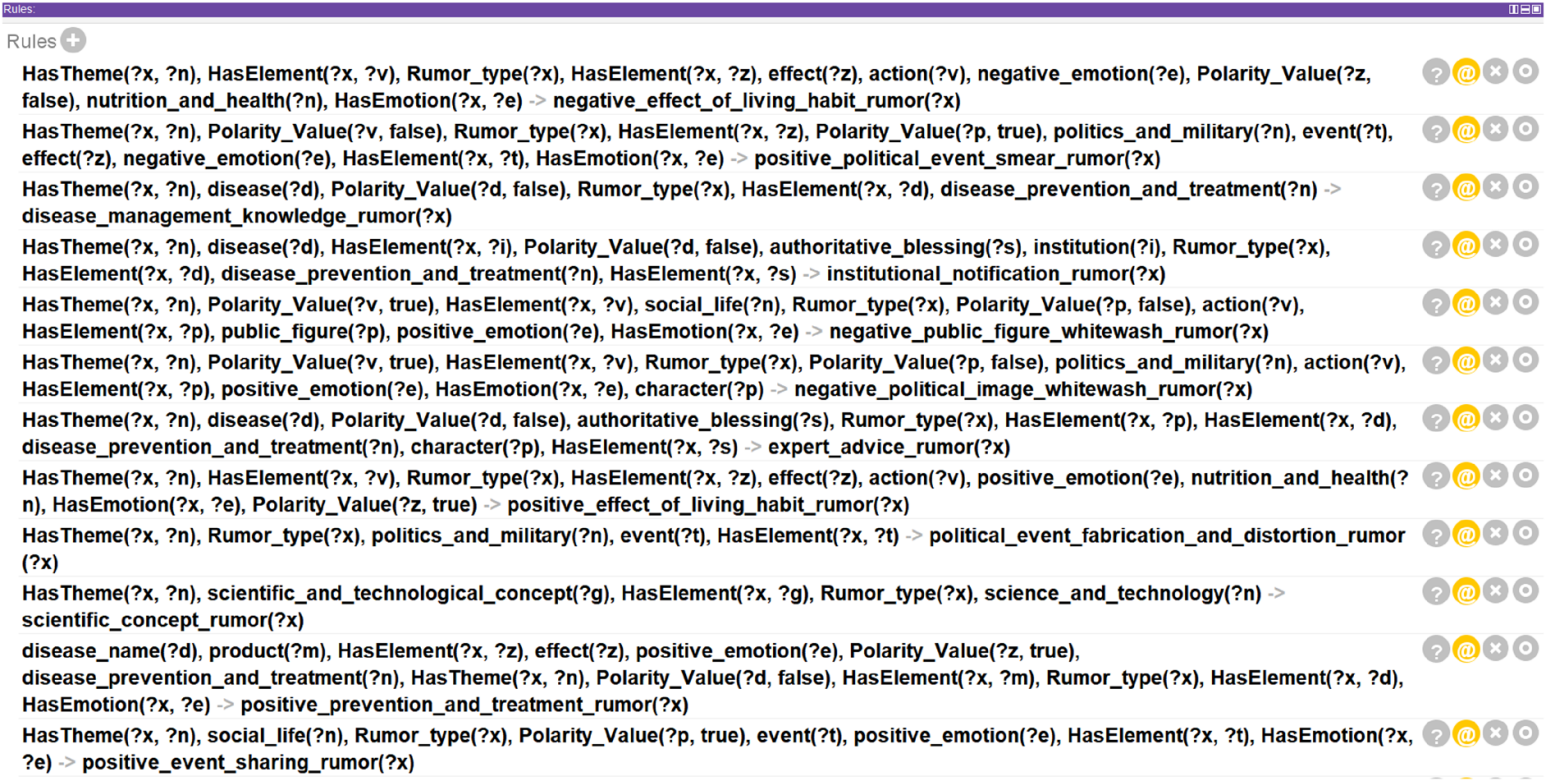
Partial SWRL rules for the rumor domain ontology.

This paper summarizes the object property features of various types of online rumors based on the self-built rumor dataset, maps the real rumor texts with the rumor domain ontology, constructs typical SWRL reasoning rules for judging 32 typical rumor types, as shown in Table 13, and imports them into the protégé rule library, as shown in Fig. 6. In which x, n, e, z, i, t, v, l, etc. are instances of rumor types, text theme, emotion category, effect, institution, event, action, geographical position, etc. in the ontology. HasTheme, HasEmotion, HasElement, HasSource, HasMood and HasSupport are object property relationships. Polarity value is a data property relationship.

**Table 13:** Partial SWRL rules for the rumor domain ontology.

Rule name	Rule expressions
Positive political image smear rumor judgment rule	rumor type(?x) ^ politics and military(?n) ^ negative emotion(?e) ^ HasTheme(?x,?n) ^ HasEmotion(?x,?e) ^ political character(?p) ^ HasElement(?x,?p) ^Polarity_Value(?p,true) ^ action(?v)^ HasElement(?x,?v) ^ Polarity_Value(?v,false)-> positive political image smear rumor(?x) ……
Negative effect of living habit rumor judgment rules	rumor type(?x) ^ action(?v) ^ nutrition and health(?n) ^ negative emotion(?e) ^ HasElement(?x,?v) ^ HasTheme(?x,?n) ^ HasEmotion(?x,?e) ^ effect(?z) ^ HasElement(?x,?z) ^Polarity_Value(?z,false)-> negative effect of living habit rumor(?x) ……
Disease management knowledge rumor judgment rules	rumor type(?x) ^ disease prevention and treatment(?n) ^ HasTheme(?x,?n) ^ disease (?d)^ HasElement(?x,?d) ^ Polarity_Value(?d,false)-> disease management knowledge rumor (?x) ……
Natural disaster occurrence rumor judgment rules	rumor type(?x) ^ social life(?n) ^ negative emotion(?e) ^ geographical position(?l) ^ natural disasters(?h) ^ HasTheme(?x,?n) ^ HasEmotion(?x,?e) ^ HasElement(?x,?l) ^ HasElement(?x,?h)-> natural disaster occurrence rumor(?x) ……
Scientific theory rumor judgment rules	rumor type(?x) ^ science and technology(?n) ^ HasTheme(?x,?n) ^ scientific theories(?q) ^ HasElement(?x,?q)-> scientific theory rumor(?x) ……

### Implicit knowledge mining and verification based on pellet reasoner

This paper extracts corresponding instances from the rumor dataset, imports the rumor domain ontology and SWRL rule description into the pellet reasoner in the protégé software, performs implicit knowledge mining of the rumor domain ontology, judges the rumor type of the instance, and verifies the semantic parsing ability and consistency of the ontology.

**Figure 7:**
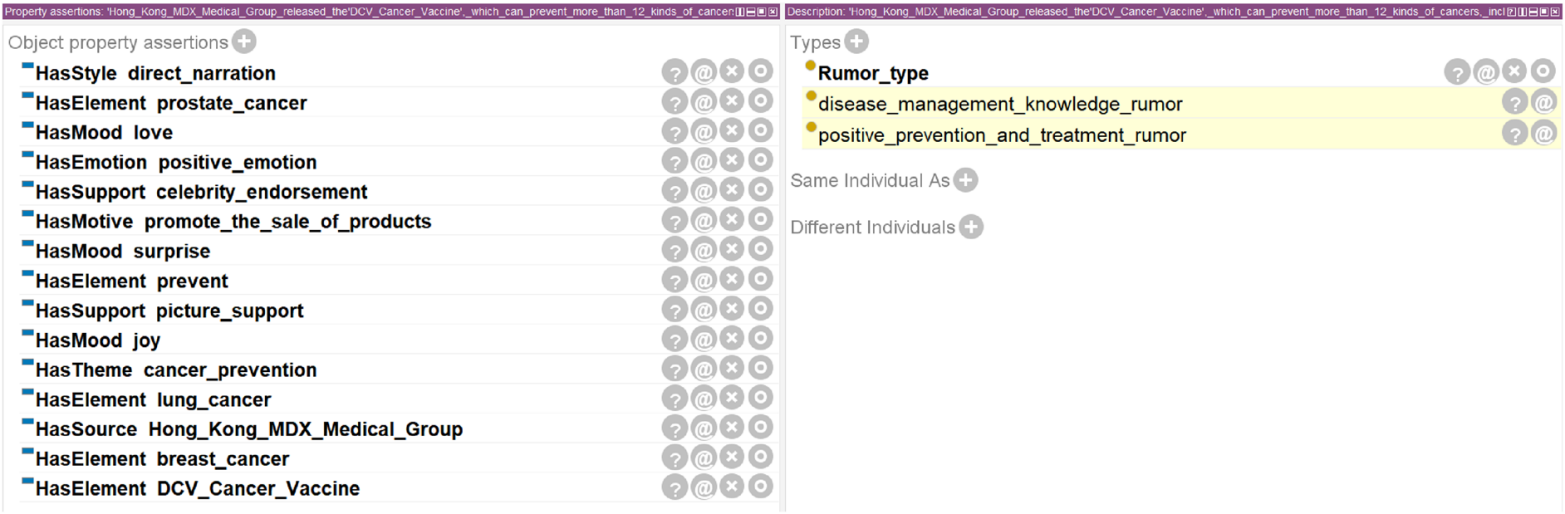
Implicit relationship between rumor instance parsing results and pellet reasoner mining.

Positive prevention and treatment of disease rumors are mainly based on the theme of disease prevention and treatment, usually containing products to be sold (including drugs, vaccines, equipment, etc.) and effect of disease names, claiming to have positive effects (such as prevention, cure, relief, etc.) on certain diseases or symptoms, causing positive emotions such as surprise and happiness among patients and their families, thereby achieving the purpose of selling products. The text features and emotional features of this kind of rumors are relatively clear, so this paper takes the rumor text “Hong Kong MDX Medical Group released the ‘DCV Cancer Vaccine’, which can prevent more than 12 kinds of cancers, including prostate cancer, breast cancer and lung cancer.” as an example to verify the semantic parsing ability of the rumor domain ontology. The analysis result of this instance is shown in Fig. 7. The text theme is cancer prevention in disease prevention and treatment, the text style is plain narrative style, and the text element includes product-DCV cancer vaccine, positive effect-prevention, disease name-prostate cancer, disease name-breast cancer, disease name-lung cancer; the emotion category of this instance is a positive emotion, emotional appeal is joy, love, surprise; The motive for releasing rumors is profit-driven in selling products, the information source is Hong Kong MDX medical group, and pictures and celebrity endorsements are used as testimony method. This paper uses a pellet reasoner to reason on the parsed instance based on SWRL rules, and mines out the specific rumor type of this instance as positive prevention and treatment of disease rumor. This paper also conducted similar instance analysis and reasoning verification for other types of rumor texts, and the results show that the ontology has high consistency and reliability.

### Comparison and evaluation of ontology performance

In this paper, the constructed ontology is compared with the representative rumor index system in the field. By inviting four experts to make a comprehensive evaluation based on the self-built index system^70–72^, their performance in the indicators of reliability, coverage and operability is evaluated. According to the ranking order given by experts, they are given 1–4 points, and the first place in each indicator item gets four points. The average value given by three experts is taken as the single indicator score of each subject, and the total score of each indicator item is taken as the final score of the subject.

As can be seen from Table 14, the rumor domain ontology constructed in this paper constructs a term set through three ways: reusing the existing ontology, extracting the content features of core documents and discovering new concepts based on real rumor data sets, and the ontology structure has been verified by SWRL rule reasoning of pellet inference machine, which has high reliability; ontology covers six kinds of Chinese online rumors, including the grammatical, semantic, pragmatic and social characteristics of rumor text characteristics, emotional characteristics, rumor credibility and social background, which has a high coverage; ontology is coded by OWL language specification and displayed visually on protege, which is convenient for further expansion and reuse of scholars and has high operability.

**Table 14:** Comparison and evaluation of ontology performance.

Indicator score	The ontology constructed in this paper	Zhou, L., Tao, J., and Zhang, D. S. (2023)^13^	Zhou, G. (2021)^10^	Yu, G. (2018)^8^
Reliability	Ontology reuse, literature review, data analysis and reasoning verification	Literature review, subject modeling analysis	Literature review, data analysis	Data analysis
	4	2.75	2	1.25
Coverage	Six kinds of Chinese internet rumors	Multilingual epidemic rumors	Chinese epidemic rumors	Three kinds of Chinese internet rumors
	4	3	1	2
Operability	OWL coding, protege visualization	Text description	Text description	Text description
	4	2	2	2
Total score	12	7.75	5	5.25

## Discussion

The construction method of TFI domain ontology proposed in this paper includes terminology layer, framework layer and instance layer. Compared with the traditional methods, this paper adopts three-dimensional data set construction method in terminology layer construction, investigates top-level ontology and related core documents, and completes the mapping of reusable top-level ontology from top to bottom and the concept extraction of rumor content features in existing literature research. Based on the mainstream internet rumor websites in China, the authoritative real rumor data set is established, and the new word discovery algorithm of N-gram combined with RoBERTa-Kmeans clustering algorithm is used to automatically discover new concepts in the field from bottom to top; determine the terminology set of domain ontology more comprehensively and efficiently. This paper extracts the clustering results of domain concepts based on the content characteristics of core documents in the selection of parent rumors content characteristics in the framework layer construction, that is, rumors text characteristics, rumors emotional characteristics, rumors credibility characteristics and social background characteristics; based on the emotional characteristics and the entity categories of real rumor data sets, the characteristics of rumor categories are defined. Sub-category rumor content features combine the concept of three-dimensional rumor term set and the concept distribution based on real rumor data set, define the sub-category concept and hierarchical relationship close to the real needs, and realize the fine-grained hierarchical modeling of the relationship between multi-domain network rumor content features. In this paper, OWL language is used to encode the rumor domain ontology in the instance layer construction, and SWRL rule language and Pellet inference machine are used to deal with the conflict and mine tacit knowledge, judge the fine-grained categories of rumor texts, and realize the effective quality evaluation of rumor ontology. This makes the rumor domain ontology constructed in this paper have high consistency and reliability, and can effectively analyze and reason different types of rumor texts, which enriches the knowledge system in this field and provides a solid foundation for subsequent credible rumor detection and governance.

However, the study of the text has the following limitations and deficiencies:

- (1) The rumor domain ontology constructed in this paper only considers the content characteristics, but does not consider the user characteristics and communication characteristics. User characteristics and communication characteristics are important factors affecting the emergence and spread of online rumors, and the motivation and influence of rumors can be analyzed. In this paper, these factors are not included in the rumor feature system, which may limit the expressive ability and reasoning ability of the rumor ontology and fail to fully reflect the complexity and multidimensional nature of online rumors.
- (2) In this paper, the mainstream Internet rumor-dispelling websites in China are taken as the data source of ontology instantiation. The data covers five rumor categories: political and military, disease prevention, social life, science and technology, and nutrition and health, and the data range is limited. And these data sources are mainly official or authoritative rumor websites, and their data volume and update frequency may not be enough to reflect the diversity and variability of online rumors, and can not fully guarantee the timeliness and comprehensiveness of rumor data.
- (3) The SWRL reasoning rules used in this paper are based on manual writing, which may not cover all reasoning scenarios, and the degree of automation needs to be improved. The pellet inference engine used in this paper is an ontology inference engine based on OWL-DL, which may have some computational complexity problems and lack of advanced reasoning ability.

The following aspects can be considered for optimization and improvement in the future:

- (1) This paper will introduce user characteristics into the rumor ontology, and analyze the factors that cause and accept rumors, such as social attributes, psychological state, knowledge level, beliefs and attitudes, behavioral intentions and so on. This paper will introduce the characteristics of communication, and analyze the propagation dynamic factors of various types of rumors, such as propagation path, propagation speed, propagation range, propagation period, propagation effect, etc. This paper hopes to introduce these factors into the rumor feature system, increase the breadth and depth of the rumor domain ontology, and provide more credible clues and basis for the detection, intervention and prevention of rumors.
- (2) This paper will expand the data sources, collect the original rumor data directly from social media, news media, authoritative rumor dispelling institutions and other channels, and build a rumor data set with comprehensive types, diverse expressions and rich characteristics; regularly grab the latest rumor data from these data sources and update and improve the rumor data set in time; strengthen the expressive ability of rumor ontology instance layer, and provide full data support and verification for the effective application of ontology.
- (3) The text will introduce GPT, LLaMA, ChantGLM and other language models, and explore the automatic generation algorithm and technology of ontology inference rules based on rumor ontology and dynamic Prompt, so as to realize more effective and intelligent rumor ontology evaluation and complex reasoning.

## Conclusion

This paper proposed a method of constructing TFI network rumor domain ontology. Based on the concept distribution of three-dimensional term set and real rumor data set, the main features of network rumors are defined, including text features, emotional features, credibility features, social background features and category features, and the relationships among these multi-domain features are modeled in a fine-grained hierarchy, including five parent classes and 88 subcategories. At the instance level, 32 types of typical rumor category judgment and reasoning rules are constructed, and the ontology is processed by using SWRL rule language and pellet inference machine for conflict processing and tacit knowledge mining, so that the semantic analysis and reasoning of rumor text content are realized, which proves its effectiveness in dealing with complex, fuzzy and uncertain information in online rumors and provides a new perspective and tool for the interpretable analysis and processing of online rumors.

## Data Availability

The datasets generated during the current study are available from the corresponding author upon reasonable request.
