# Author Correction: Research on domain ontology construction based on the content features of online rumors

**DOI:** 10.1038/s41598-024-64754-6

**Published:** 2024-06-18

**Authors:** Jianbo Zhao, Huailiang Liu, Weili Zhang, Tong Sun, Qiuyi Chen, Yuehai Wang, Jiale Cheng, Yan Zhuang, Xiaojin Zhang, Shanzhuang Zhang, Bowei Li, Ruiyu Ding

**Affiliations:** 1 School of Economics and Management, Xidian University, 266 Xifeng Road, Xi’an, 710071 China; 2 School of Artifcial Intelligence, Xidian University, 266 Xifeng Road, Xi’an, 710071 China; 3 School of Telecommunications Engineering, Xidian University, 266 Xifeng Road, Xi’an, 710071 China

Correction to: *Scientific Reports* 10.1038/s41598-024-62459-4, published online 27 May 2024

The original version of this Article contained an error in Figure 1 where the wrong version was typeset. The original Figure 1 and accompanying legend appear below.

**Figure 1:**
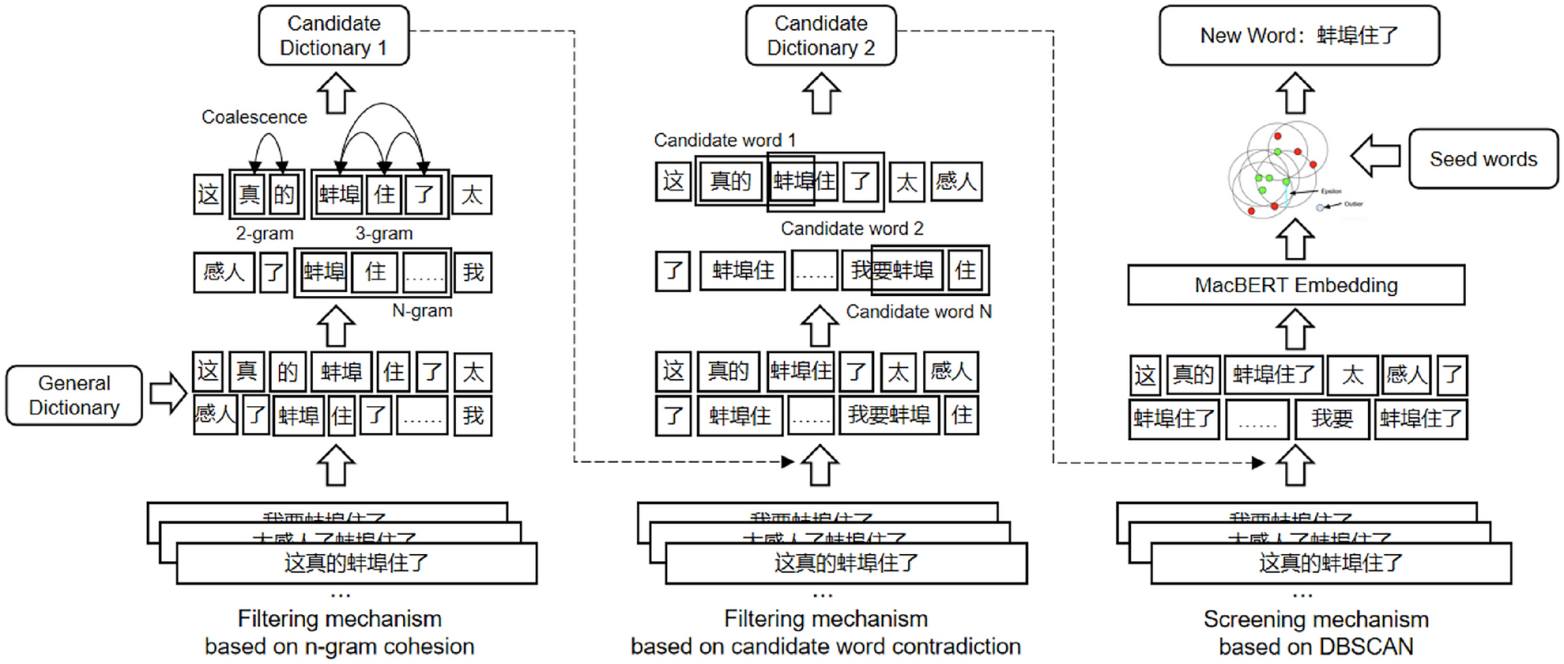
Flowchart of domain new word discovery algorithm.

The original Article has been corrected.

